# Supplementary figures and images for: Inferring evolutionary pathways and directed genotype networks of foodborne pathogens
Source: PLoS Comput Biol. 2020 Oct 30;16(10):e1008401. doi: 10.1371/journal.pcbi.1008401 (PMC7657559; doi:10.1371/journal.pcbi.1008401)

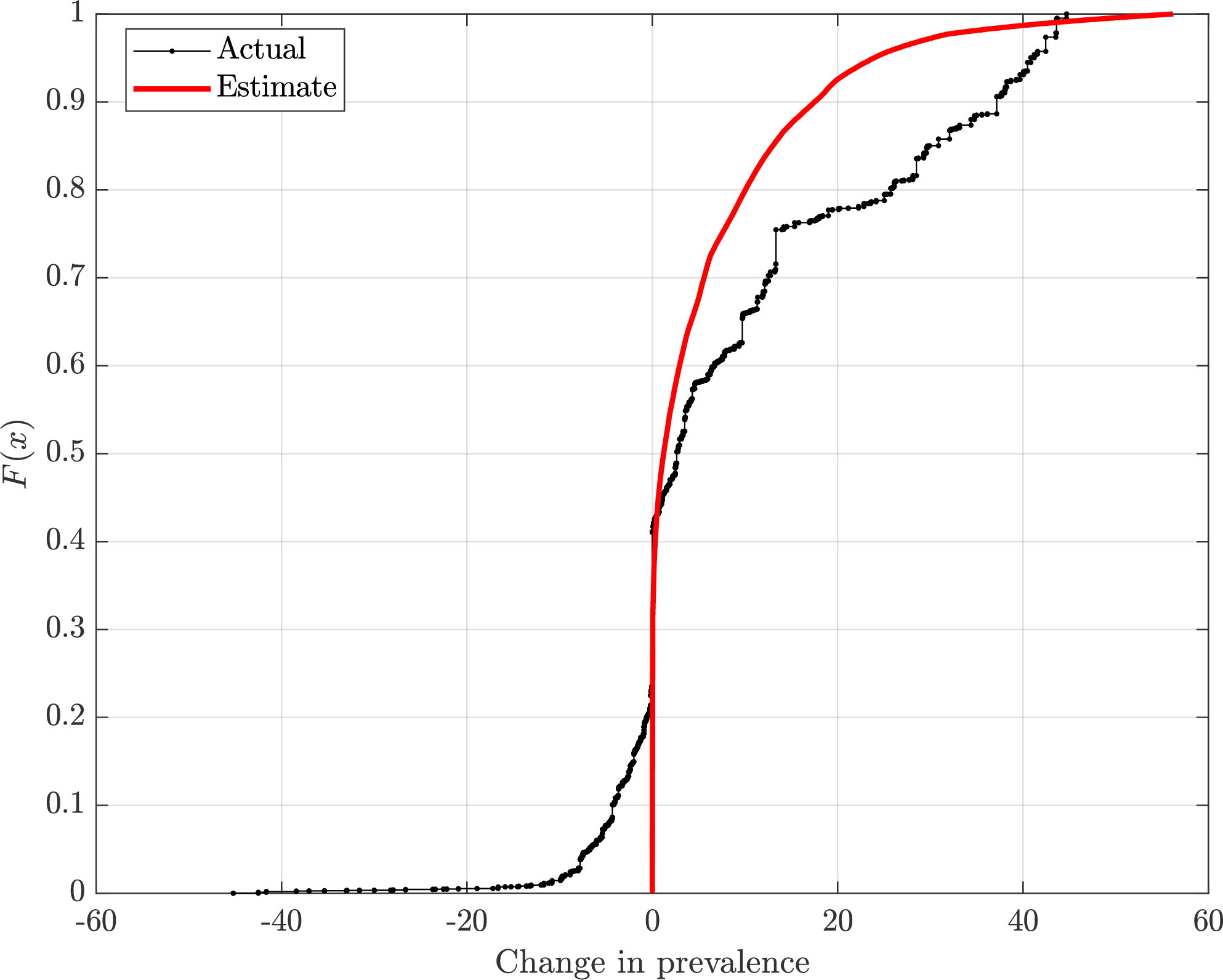

Supplement: S1 Fig — The contributions of all unsuccessful paths are bounded to 1. (TIF) [file pcbi.1008401.s001.tif]

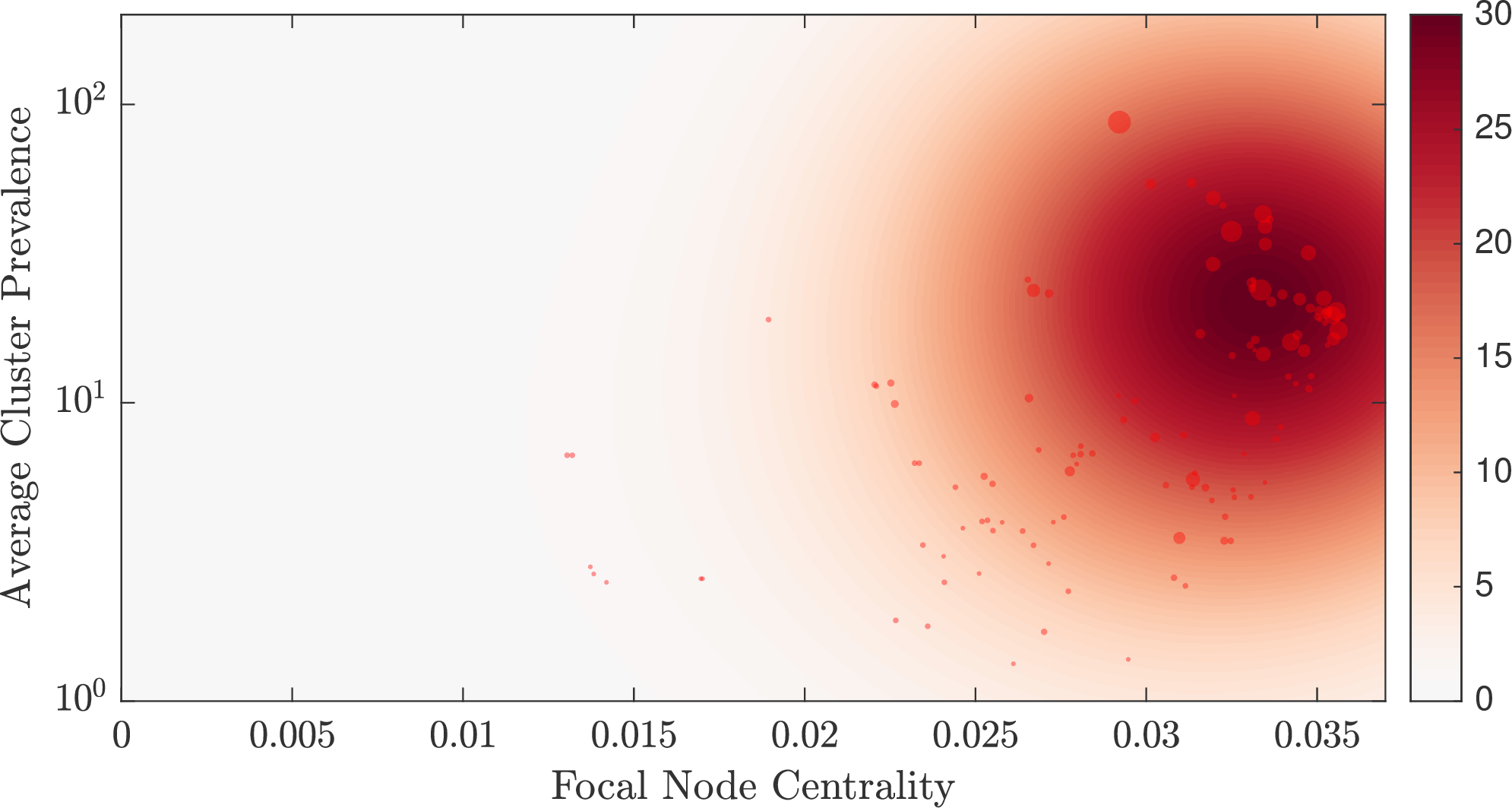

Supplement: S2 Fig — The structure of the centrality-prevalence space, revealed by the expected value of only successful evolutionary paths. For every MLVA profile, the point size is shown in proportion to the average (positive) change in prevalence. Colour intensity indicates the estimated density, given the average positive changes in prevalence. Similar to Fig 5, the transition region (intense red colour) is shaped by the successful profiles with high centrality and medium cluster prevalence. The evolutionary paths originating from profiles in this region tend to develop from right to left and from bottom to top, producing the higher average change in prevalence. (TIF) [file pcbi.1008401.s002.tif]

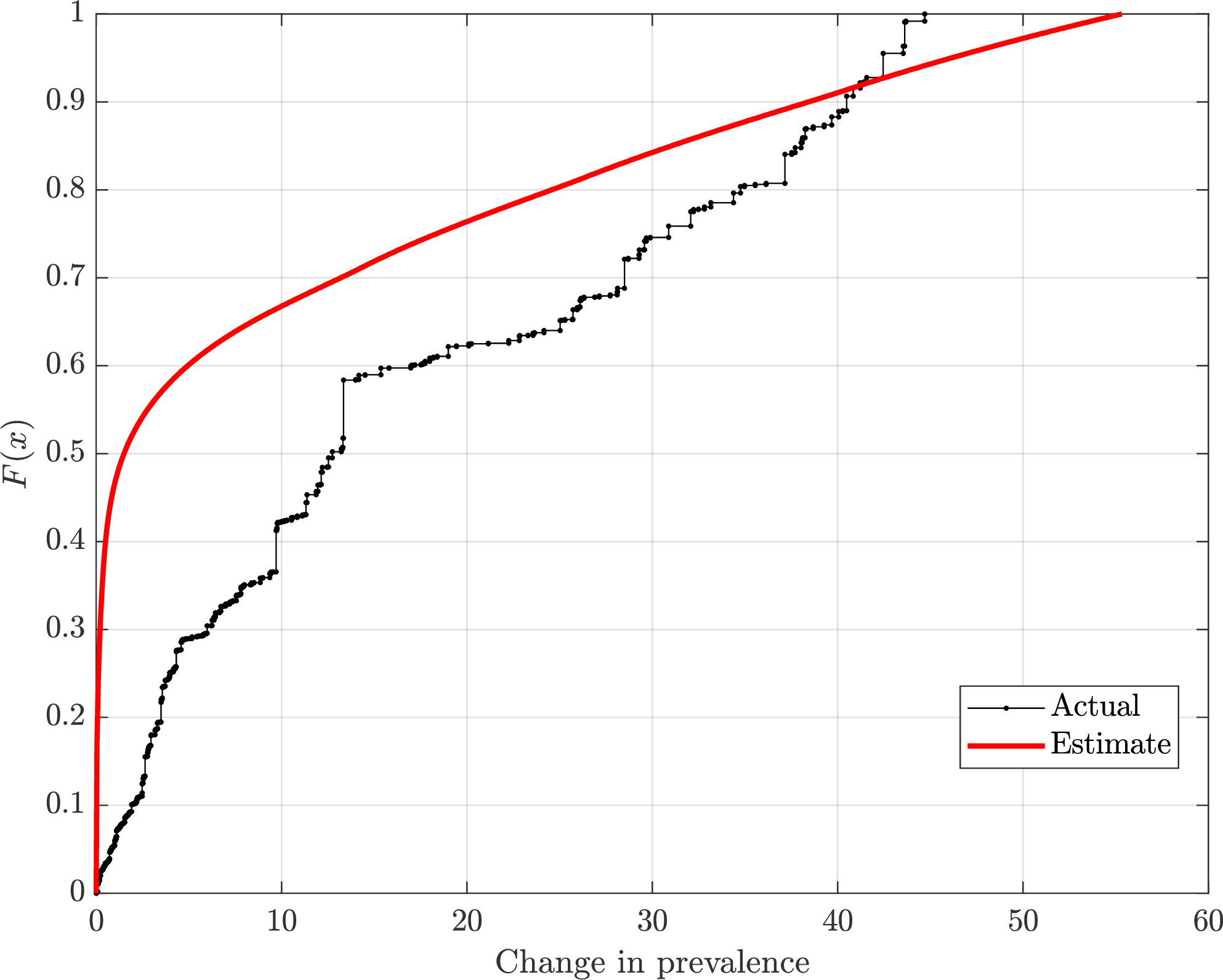

Supplement: S3 Fig — (TIF) [file pcbi.1008401.s003.tif]

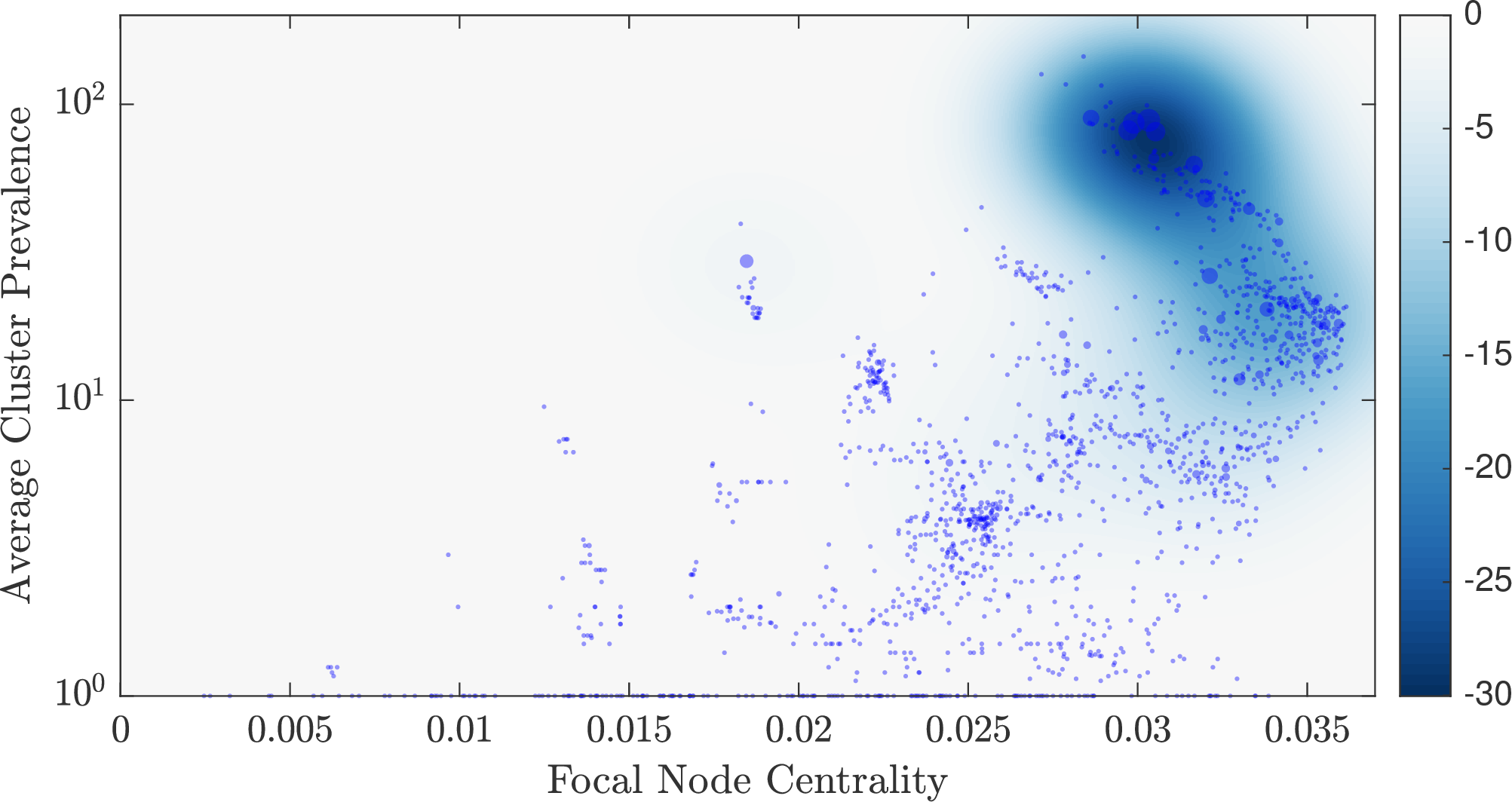

Supplement: S4 Fig — The structure of the centrality-prevalence space, revealed by the expected value of only unsuccessful evolutionary paths. For every MLVA profile, the point size is shown in proportion to the average (negative) change in prevalence. Colour intensity indicates the estimated density, given the average negative changes in prevalence. Similar to Fig 5, the bottleneck region (intense blue colour) is formed by unsuccessful profiles with the centrality around 0.03 and the cluster prevalence just below 102. The evolutionary paths originating from these profiles tend to develop from left to right and from top to bottom, reducing their prevalence on average. (TIF) [file pcbi.1008401.s004.tif]

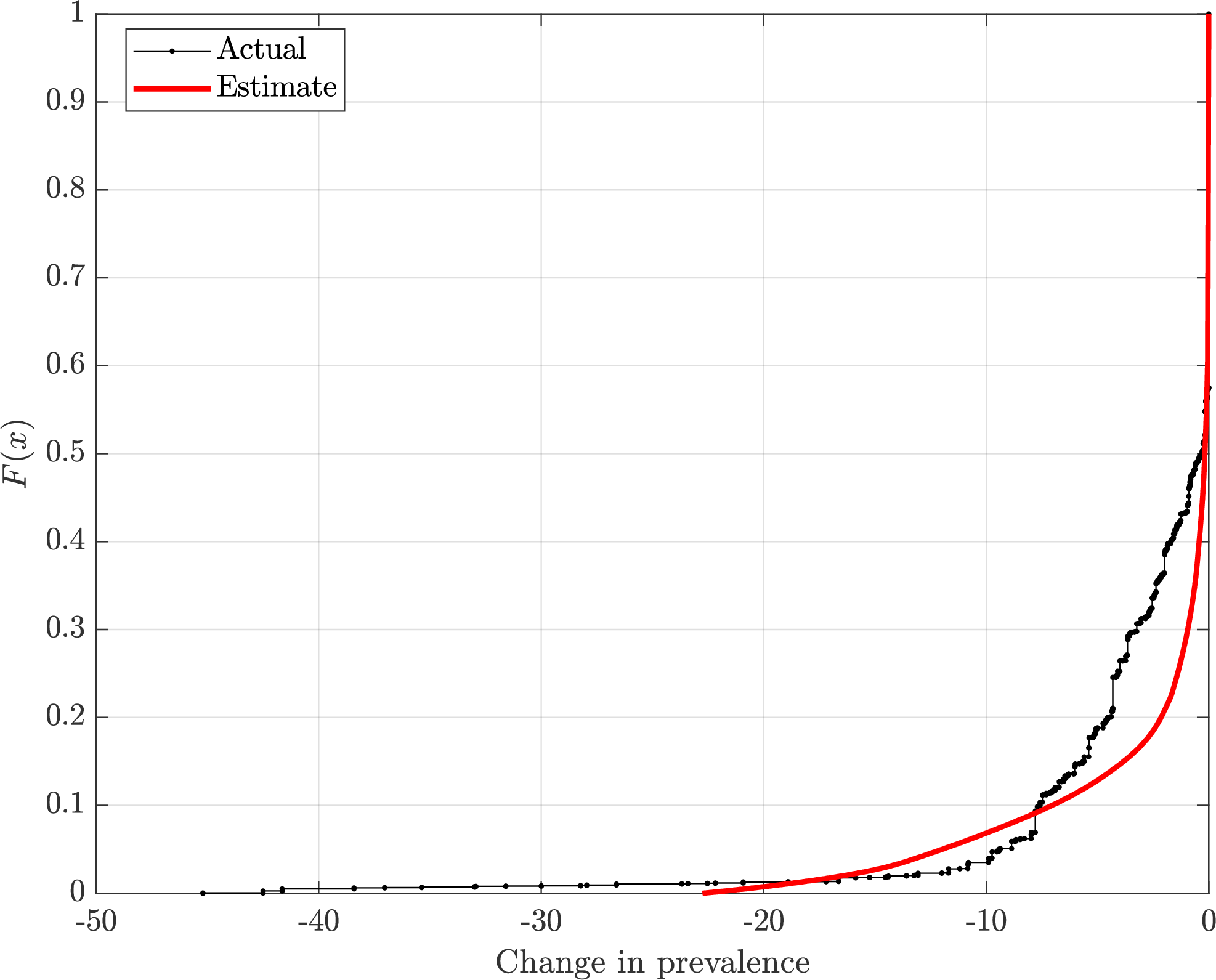

Supplement: S5 Fig — (TIF) [file pcbi.1008401.s005.tif]

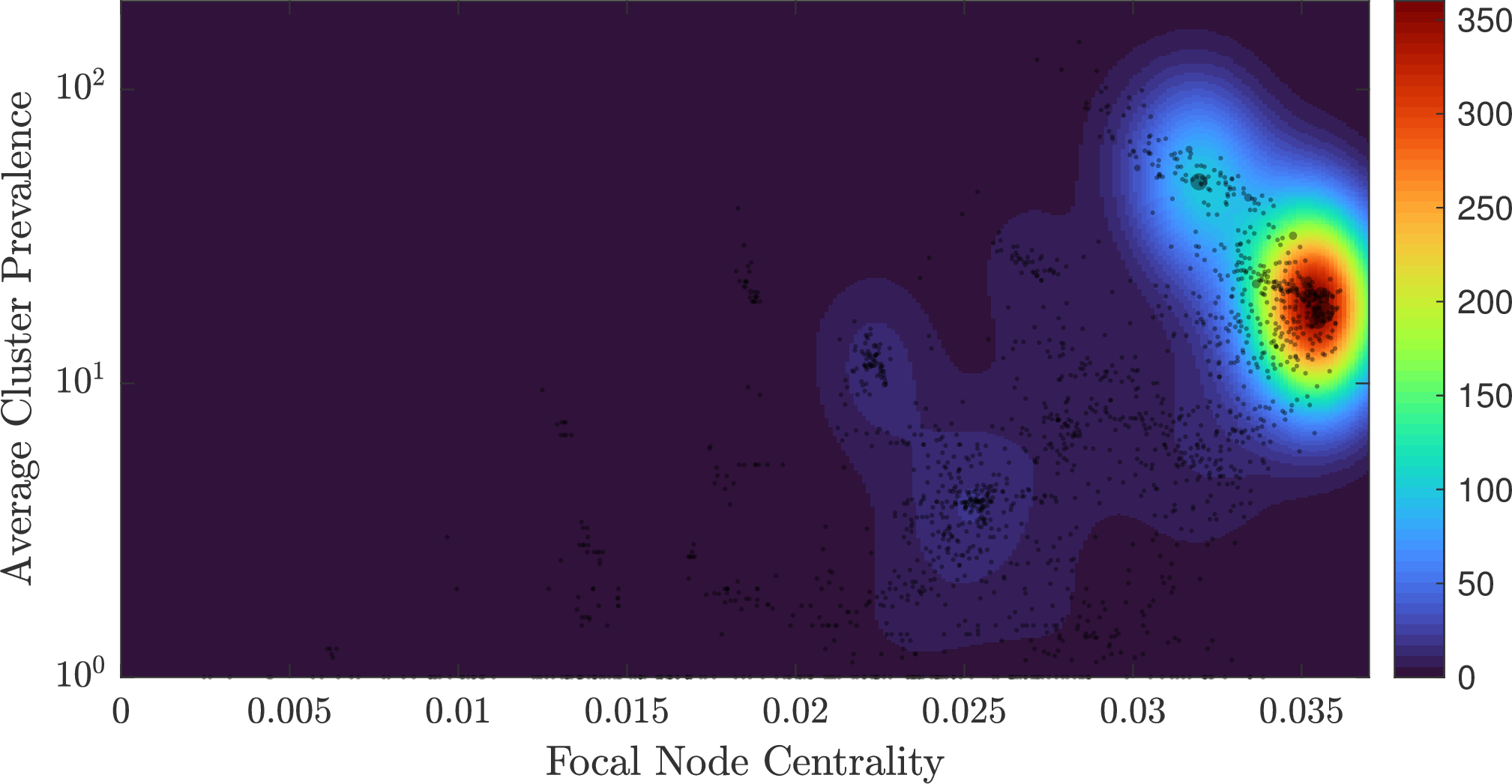

Supplement: S6 Fig — The structure of the centrality-prevalence space, revealed by the expected number of evolutionary paths. For every MLVA profile, the point size is shown in proportion to the number of paths that start from this profile. Colour intensity indicates the estimated density. (TIF) [file pcbi.1008401.s006.tif]

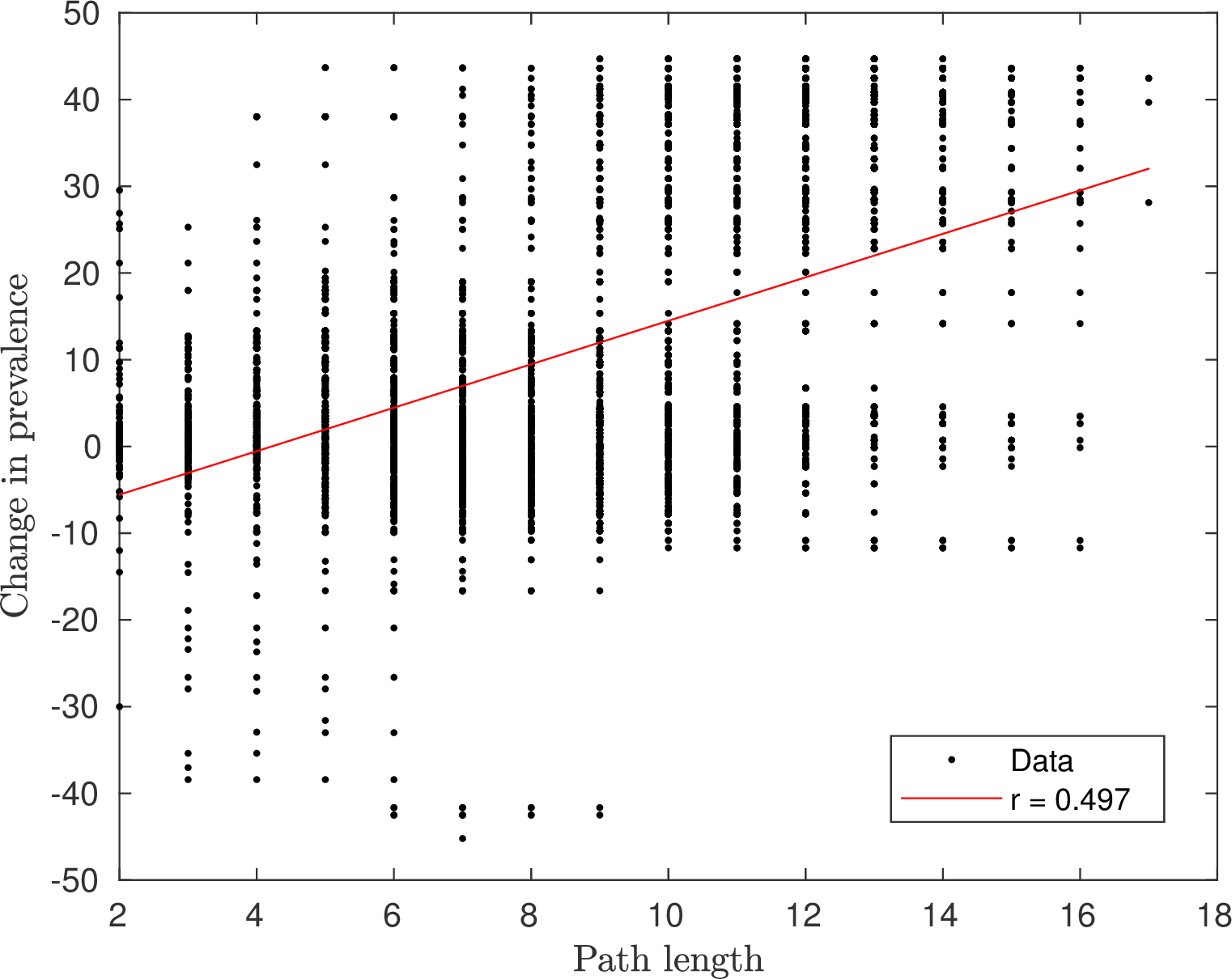

Supplement: S7 Fig — Correlation between the path length and the change in the average prevalence, measured as r = 0.497 (N = 6, 897, p < 0.00001). (TIF) [file pcbi.1008401.s007.tif]

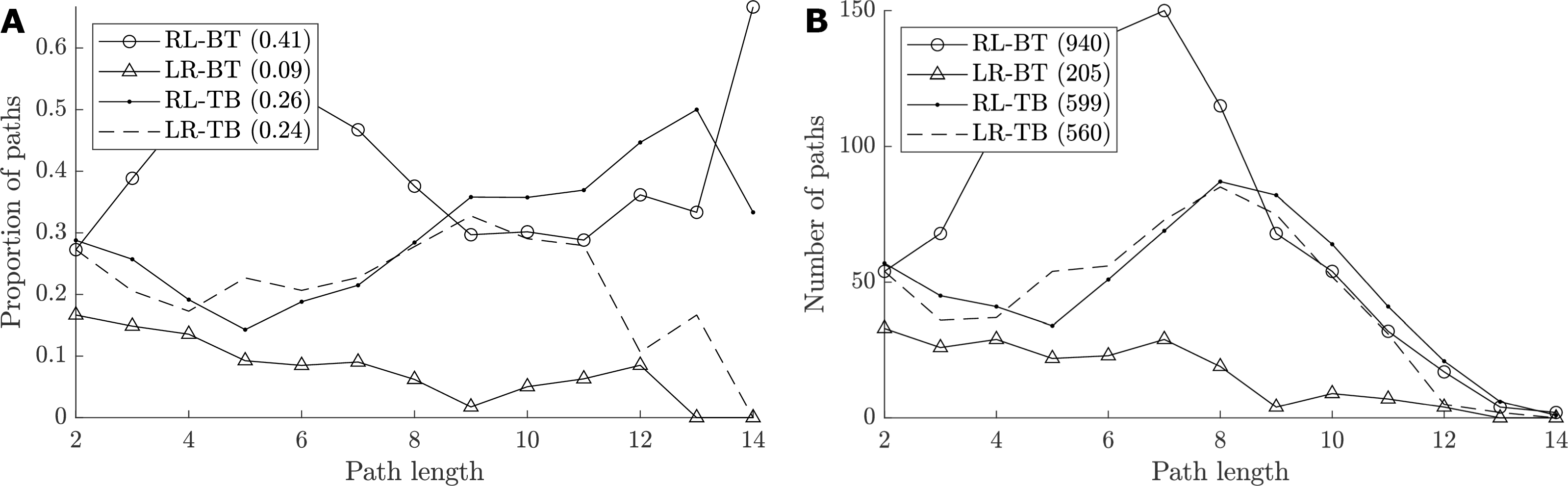

Supplement: S8 Fig — Constrained to genetic variations within one locus only. Number of nodes N = 718, number of edges is 967, and the number of paths M = 2, 304. Correlation of path length to change in prevalence: r = −0.121 (M = 2, 304, p < 0.00001). (TIF) [file pcbi.1008401.s008.tif]

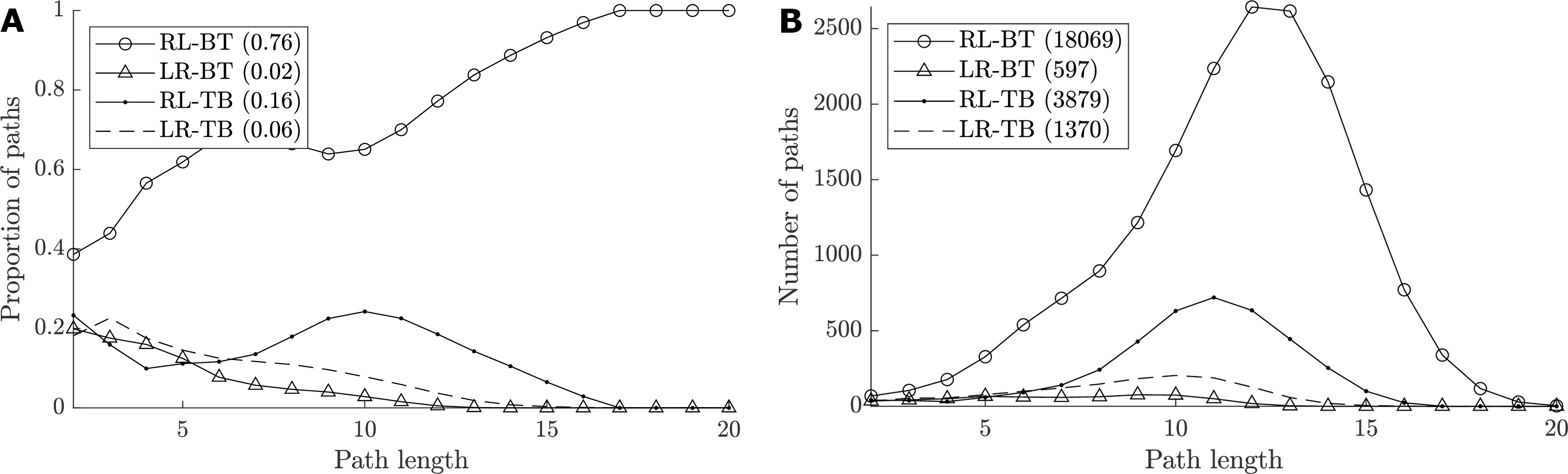

Supplement: S9 Fig — Constrained to genetic variations over multiple loci with Larsson’s correction. Number of nodes N = 744, number of edges is 1,190, and the number of paths M = 23, 915. Correlation of path length to change in prevalence: r = 0.269 (M = 23, 915, p < 0.00001). (TIF) [file pcbi.1008401.s009.tif]

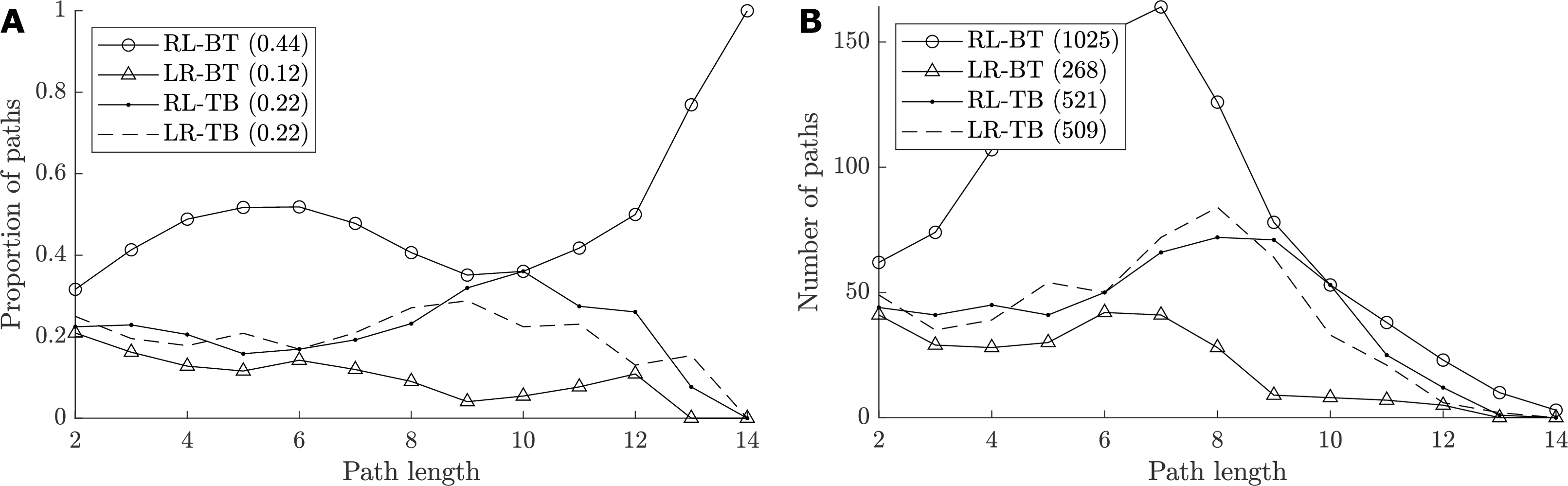

Supplement: S10 Fig — Constrained to genetic variations within one locus only with Larsson’s correction. Number of nodes N = 730, number of edges is 991, and the number of paths M = 2, 323. Correlation of path length to change in prevalence: r = −0.0474 (M = 2, 323, p = 0.0224). (TIF) [file pcbi.1008401.s010.tif]

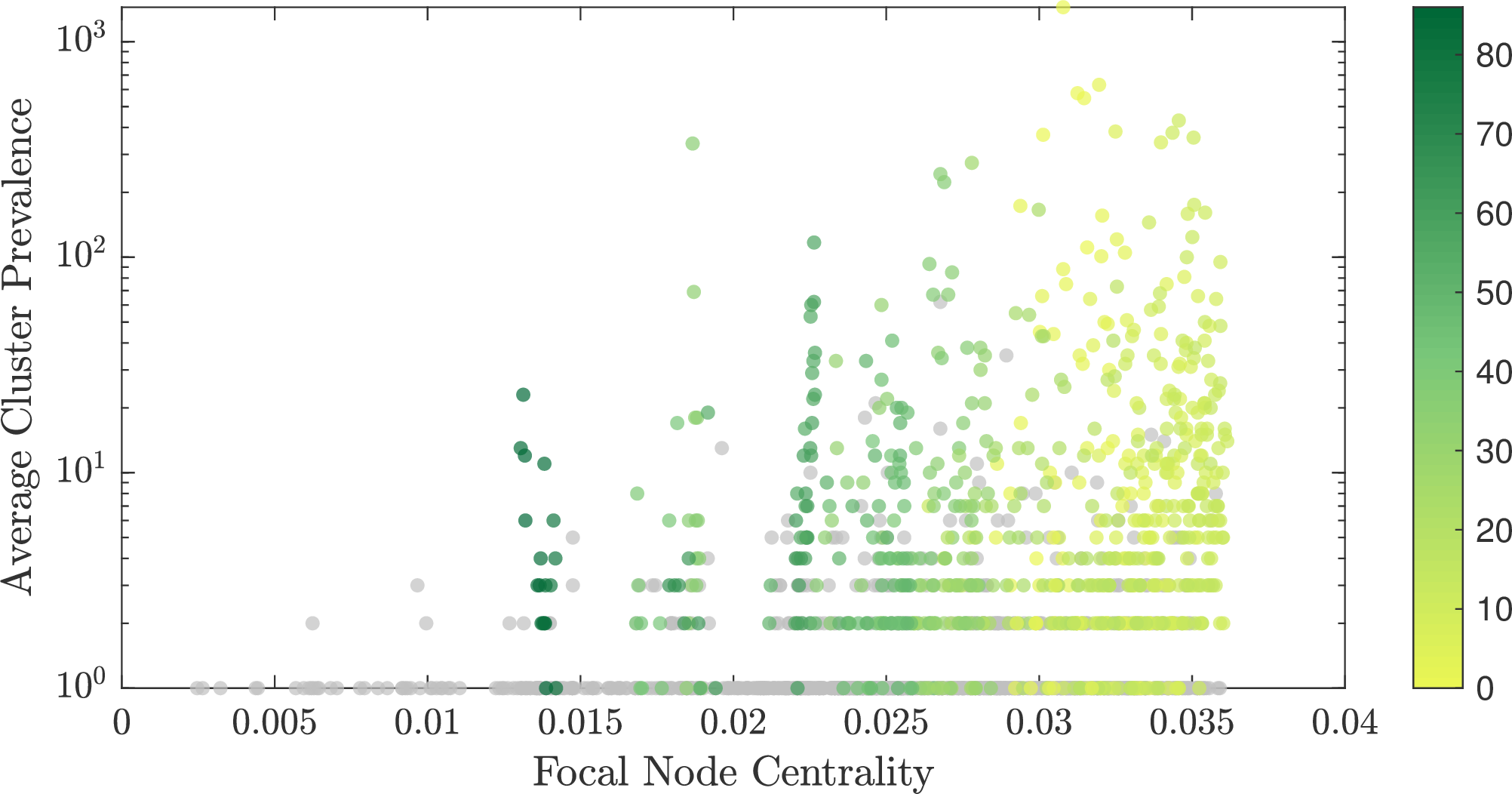

Supplement: S11 Fig — The centrality-prevalence space for clustering threshold Gmax = 0 (singleton-clusters). The centrality is measured with respect to the undirected genotype network. The colour shows the genetic distance of a profile to the reference profile (the most prevalent node), with grey points indicating disconnected profiles in the directed network. Correlation between the distance to the most prevalent node and the log-prevalence r = −0.143 (N = 690, p = 1.62 ⋅ 10−4). Correlation between the log-component-size and log-prevalence: r = 0.332 (N = 690, p < 0.00001). (TIF) [file pcbi.1008401.s011.tif]

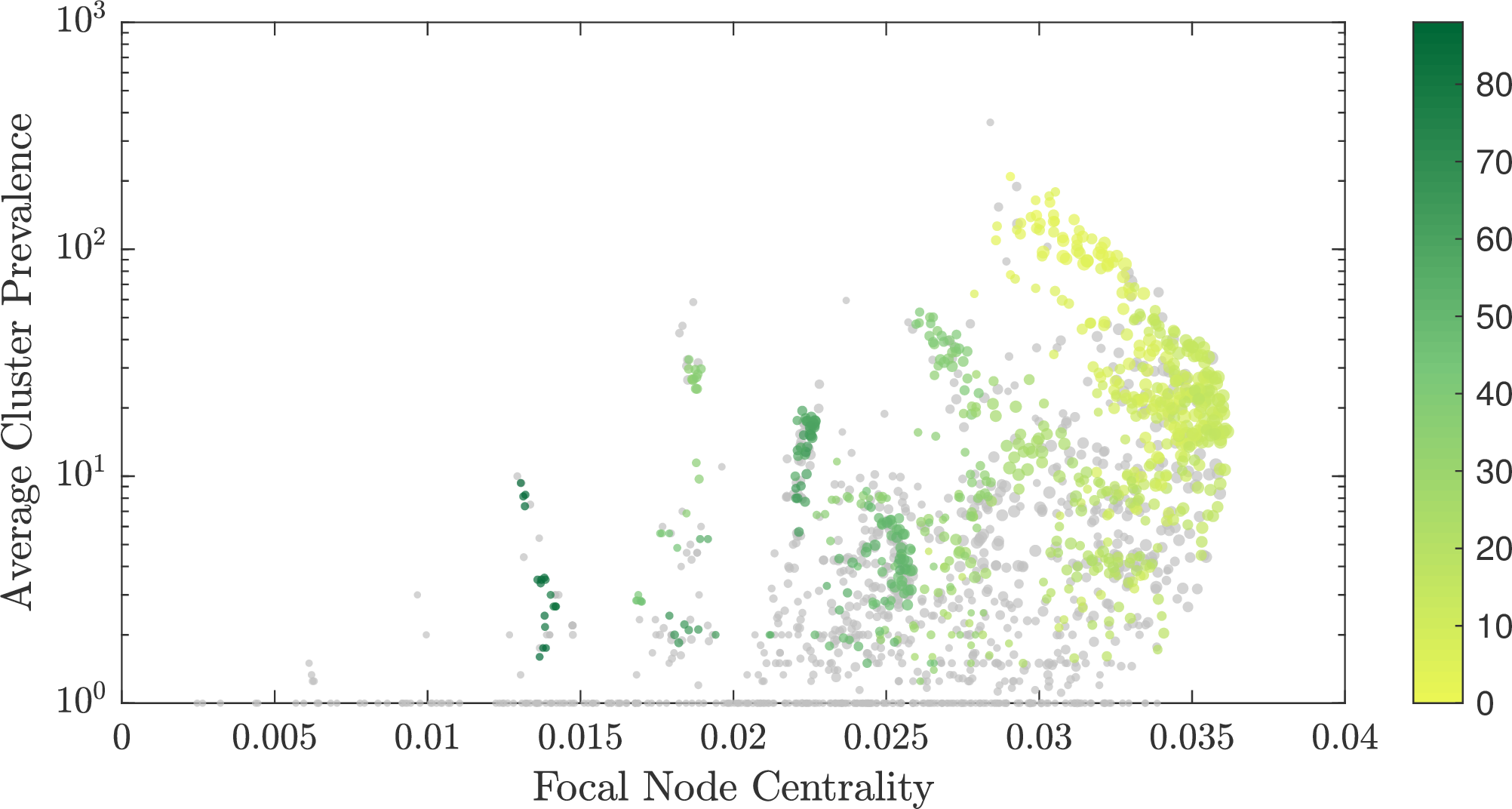

Supplement: S12 Fig — The centrality-prevalence space for clustering threshold Gmax = 3. The centrality is measured with respect to the undirected genotype network. The colour shows the genetic distance of a profile to the reference profile (the most prevalent node), with grey points indicating disconnected profiles in the directed network. Correlation between the distance to the most prevalent node and the log-prevalence r = −0.513 (N = 690, p < 0.00001). Correlation between the log-component-size and log-prevalence: r = 0.734 (N = 690, p < 0.00001). (TIF) [file pcbi.1008401.s012.tif]

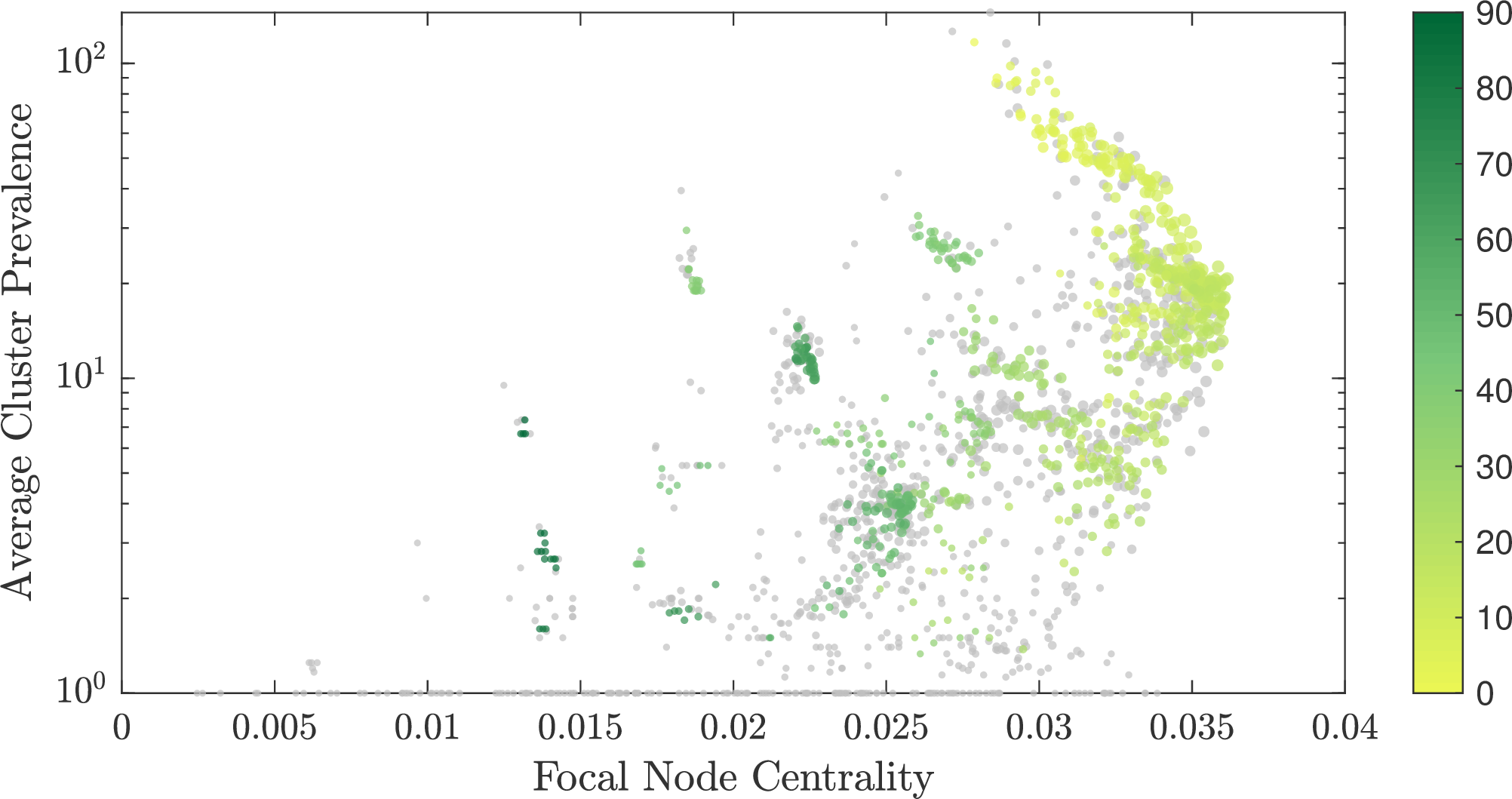

Supplement: S13 Fig — The centrality-prevalence space for clustering threshold Gmax = 5. The centrality is measured with respect to the undirected genotype network. The colour shows the genetic distance of a profile to the reference profile (the most prevalent node), with grey points indicating disconnected profiles in the directed network. Correlation between the distance to the most prevalent node and the log-prevalence r = −0.613 (N = 690, p < 0.00001). Correlation between the log-component-size and log-prevalence: r = 0.739 (N = 690, p < 0.00001). (TIF) [file pcbi.1008401.s013.tif]

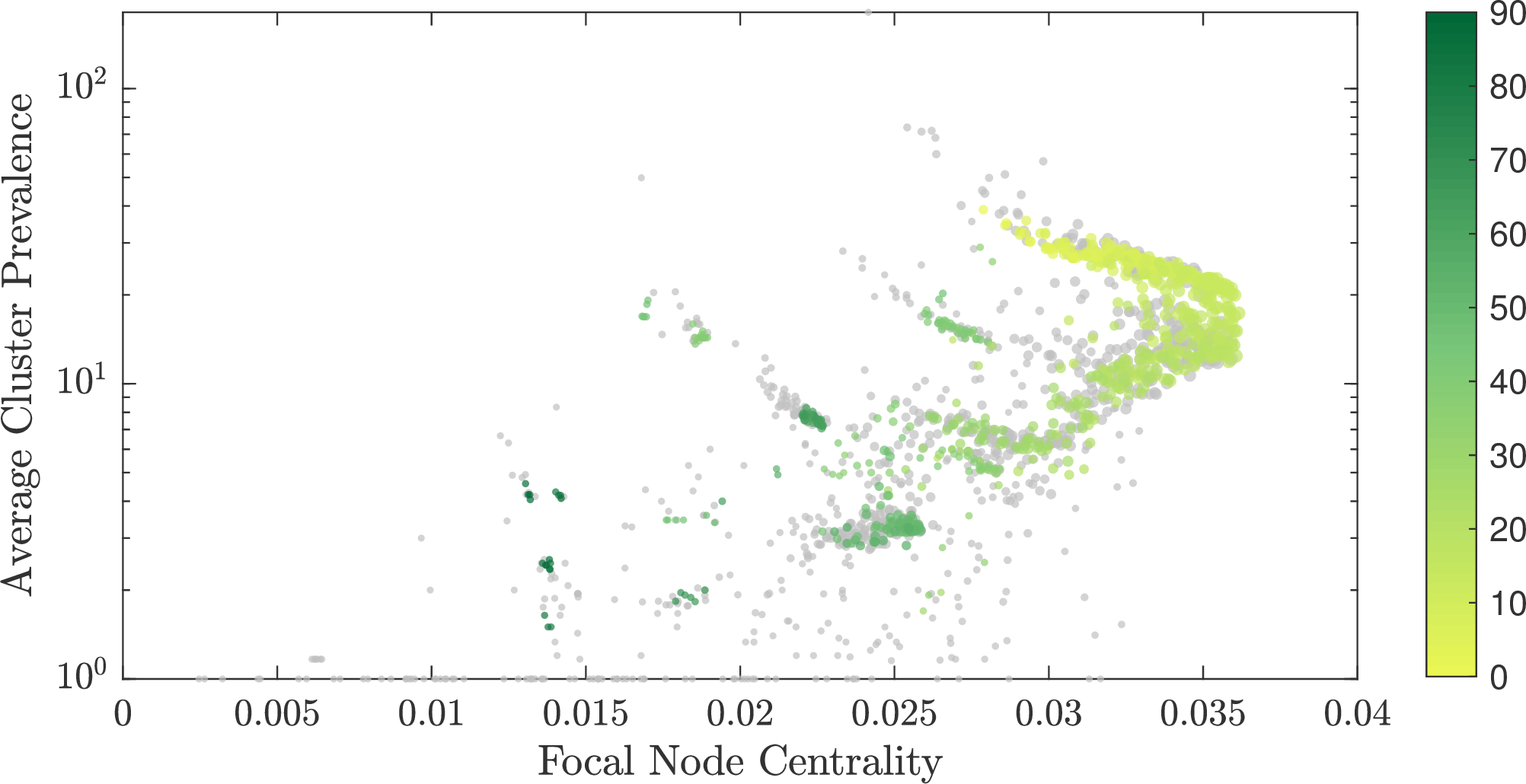

Supplement: S14 Fig — The centrality-prevalence space for clustering threshold Gmax = 10. The centrality is measured with respect to the undirected genotype network. The colour shows the genetic distance of a profile to the reference profile (the most prevalent node), with grey points indicating disconnected profiles in the directed network. Correlation between the distance to the most prevalent node and the log-prevalence r = −0.795 (N = 690, p < 0.00001). Correlation between the log-component-size and log-prevalence: r = 0.683 (N = 690, p < 0.00001). (TIF) [file pcbi.1008401.s014.tif]
