# Supplementary material for: Inferring evolutionary pathways and directed genotype networks of foodborne pathogens
Source: PLoS Comput Biol. 2020 Oct 30;16(10):e1008401. doi: 10.1371/journal.pcbi.1008401 (PMC7657559; doi:10.1371/journal.pcbi.1008401)
Supplement: Attachment — Submitted filename: Response 27-Aug-2020.pdf [file pcbi.1008401.s015.pdf]

James Lloyd-Smith
Associate Editor
PLOS Computational Biology

27 August 2020

RE: PCOMPBIOL-D-20-00724

We would like to thank you and the referees for reviewing our paper and providing constructive criticism and helpful comments. Please find attached a new version of the manuscript, revised according to the reviewers' suggestions, with the modifications marked in red (except for minor grammatical edits). Our specific point-by-point responses are below.

Reviewer #1

> *Overall, this study is an improved version of the published analysis by the authors (ref 18 in the manuscript), by using Bayesian inference to build the directed genotype network. The approach is quite nice and the results provide interesting insight about the evolution of STM. As described in the discussion, the approach developed in this study may be extended to analyze datasets other than MLVA profiles, such as genome sequencing data.*

Response: We thank the reviewer for this positive feedback.

1. *Is the MLVA dataset described in this study publicly available?*

Response: Yes, we have added a data and code availability statement (lines 532-534), with the code and data used in this study being available at GitHub Digital Repository:
https://github.com/olivercliff/inferring-networks-salmonella

2. *Is the undirected network described in this study identical to the one described in the previous study (ref 18 in the manuscript)? Please clarify.*

Response: We clarified the difference, stating (lines 442-443) that our previous work [25] used the MLVA representation with Larsson's correction, while here we consider both variants: the original representation and Larsson's correction. We also removing some incomplete data points which reduced the number of isolates from 17,107 to 17,005, making the undirected network slightly different even for the MLVA representation with Larsson's correction shared by both studies.

3. *Can the author highlight the reference node in Figure 1? It will be interesting to know when the most prevalent genotype appears in time.*

Response: The reference MLVA is detected first on 3 Dec 2010, however, we did not highlight this specifically in Fig. 1, as this MLVA becomes most prevalent only on average, as we explain now, with a similar example, in Materials and Methods (lines 466-472).

4. *In line 223: It is unclear to me how the author defined "a typical evolutionary path". Was it simply based on decreasing genetic distance from the reference node (color gradient)? It seems to me the claims in lines 223 to 231 about "a typical evolutionary path" were based on the analysis of undirected network without using the information from the directed network?*

Response: We have now provided the explicit definitions of paths, as well as successful/unsuccessful and exploitative/explorative evolutionary paths on pp. 8-9.

5. *In lines 263-265: "Furthermore, there is a significant correlation between the path length and the change in the average prevalence, measured as r = 0.498 (N = 6,896, p < 0.00001)." Can the author provide a figure of this analysis for visualization?*

Response: New Fig. 15 is added in SI.

6. *In lines 267-269: "As Figure 4 illustrates, this is not the case for three other possible directions, including the "explorative" branch pointed towards both higher prevalence and higher centrality (LR-BT)." Can the author elaborate a bit more on this point? It is not clear to me how the author arrives to the conclusion that the "explorative" branch pointed towards LR-BT.*

Response: We have provided an additional analysis (p. 11) of this issue, describing two specific regions: the transition and bottleneck regions (see also new Figures 4 and 5, as well as new Figures 10, 12 and 14 in SI). We have also estimated the fractions of paths originating in the transition region specifically (p. 12), and commented on explorative paths (line 321).

7. *The three case studies were mentioned in the first paragraph of the result section, but were not described later on in the text. Is there anything being learned from the case studies?*

Response: We have now explained these cases studies in more detail (lines 323 – 334, p. 13).

8. *Legend of Figure 3 may need to be organized a bit? There is a paragraph of figure legend under each panel of Figure 3. But there is also another paragraph of figure legend at the bottom of the page.*

Response: Old Fig. 3 is now split into new Figures 3 and 6, and the legends are modified accordingly.

Reviewer #2

The paper was for the most part well written, and the research presented is interesting and nicely scoped, investigating a small but specific set of questions with applications to better controlling and understanding epidemics.

Response: We thank the reviewer for this positive feedback.

Abstract

The phrase structural "drivers" is perhaps misleading, because it's not clear that the network the authors construct is driving anything, so much as it is a reflection of other processes (e.g. strain isolation and genetic relatedness) and a useful mathematical tool for diagnosing patterns in those phenomena.

Response: The word "drivers" is now replaced by "features".

Many of the results summarized in the abstract in reference to network features whose underlying meanings are undefined. Please briefly explicitly state what the network construction is so that readers of the abstract can understand the results without reading the methods. For example, "We construct networks where nodes represent strains and directed edges represented strain evolutionary paths" or similar.

Response: The sentence has been reworded, as suggested.

> *Does component size refer to the connected-component size?*

Response: Yes, and this term is now used throughout the text.

> *Is outbreak severity a measured or calculated characteristic?*

Response: Outbreak severity is determined as average strain prevalence, and this is now specified in the abstract and the text (line 40).

> *As far as I can tell, the research as presented later doesn't show that this 66% of chains that decrease in centrality while increasing in prevalence are in the region of centrality-prevalence space that's "past" the transition point. The authors should clarify this in the text.*

Response: Yes, we have now included additional results: abstract and p. 12, supported by a new analysis of the transition region (see also new Figures 4 and 5, as well as Figures 10, 12 and 14 in SI). In short, 72.4% of all paths originate from the transition region (described explicitly), and 64% of the paths starting in this region follow the RL-BT direction, amounting to 46.3% of all paths.

> *("longer evolutionary chains result in genetically rare and virulent strains, which mostly evolve from a single transition point") I don't believe this is discussed in the manuscript, but I may have missed it. The authors should clarify this in the text.*

Response: The additional analysis mentioned in the previous point elucidated this question further, and we have now also commented (line 157) that the average cluster prevalence approximates the average virulence across genetically close strains, as well as placed this in the context of our case studies (line 273).

Author Summary ("radically new")

Response: Modified as suggested (p. 2).

Introduction

> *3-4: Add references*

Response: Added references (line 4).

> *12-17: Add references*

Response: Added references (lines 15, 18).

> *38-40: The way this is written makes it sound like networks are used to characterize the severity/prevalence of epidemics, when so far as I can tell, the network attributes are only correlated against the severity/prevalence, which are observed/measured independent of the networks. The authors should clarify this in the text.*

Response: Rephrased as "related" (line 39).

> *41: The transition point the authors refer to needs to be explicitly identified in the text and/or figures.*

Response: The transition point is now replaced by transition region, explicitly defined on p. 11, following the additional analysis.

Emergence and evolution of dominant strains

> *54: is fitness one example of function or are the authors saying these are interchangeable terms? (if they are interchangeable this should be "i.e.")*

Response: Rephrased as “i.e.” here and in other places.

> *63-66: (“Thus, our specific aim is to identify distinct evolutionary pathways within the STM population, and show that seasonal epidemics are associated with emergence of dominant STM strains, rather than with random sets of co-circulating STM genotypes [24,25].”) To make sure I understand this, is this another way to say this? -> The goal is to show that the most prevalent strains are also most network-central? (as shown to some degree later in the manuscript) Does dominant here mean most common or evolutionarily central?*

Response: Rephrased in terms of an evolutionary drive (lines 64-65). We do not mean that the most prevalent strains are also most network-central, but rather that there is a transition region at the high-centrality space, from which the evolutionary pathway reduces centrality of more prevalent nodes.

> *86-92: Add references.*

Response: Added references (line 90).

> *97-102: Add references to the source of the data. Is this where the quantitative prevalence data used in this work came from too? The manuscript could benefit from a short description of how prevalence was quantified for this dataset, since it's key to the manuscripts conclusions.*

Response: Added references (lines 92-93). The prevalence quantification is now explained on lines 105-106. Also, the data (and code) used in this study are now released at GitHub digital repository: https://github.com/olivercliff/inferring-networks-salmonella

> *105: (new MLVA profiles are detected regularly) should be "were"*

Response: Rephrased as “were” (line 108).

Model

> *149-150: What does it mean to have a heavy-tailed distribution of STM strains? Please clarify what metric this refers to (number of isolates of each strain? degree distribution of all strains?) Please also give a brief (1 or 2 sentence) explanation of how the clustering was done (feel free to reference section of methods where), and a more in-depth explanation of why the clustering was needed. What are the consequences or expected consequences of the analysis or results if clustering isn't implemented?*

Response: The frequency distribution of STM strains is now explained in lines 153-154). The clustering is elaborated on lines 156-159, with references to Materials and Methods (p. 18). The effects of clustering with different thresholds are now traced explicitly, as detailed in lines 335 - 342 (see also new Figures 19 – 22 in SI).

> *163: Should be "pathway"*

Response: Fixed.

Results

> *201-202: Similar comment to what I made in figure 2--what happened to the other 1000 or so profiles in the network?*

Response: This is now explained on line 219, specifying the number of disconnected profiles.

> *204-205: Why is prevalence averaged only over neighboring profiles?*

Response: This is now explained when discussing Clustering, as mentioned in the points addressed above: lines 156-159, with references to Materials and Methods (p. 18), in the context of approximating the average virulence across genetically close strains (that is, across various clonal complexes or groups).

> *210-221: The manuscript talks about "two well-defined regions of space" in terms of genetic branches, but I do not believe these are well defined at all. What do the authors mean by this? I eventually took this to mean that the authors are referring to all the points on Fig 3a below ~10 avg. cluster prevalence to be one branch, and all the point above ~ 10 to be the other branch, but this is very ambiguous as written. I would recommend that since the "branches" as described play a key role in the results, they should be highlighted in any figures they are visible in. Same with the plateaus that are later described. Additionally, it's not evident to the reader just from the green-gradient that there is an evolutionary pathway present in either the lower or upper branch, if I'm understanding the branches correctly. The authors should refine how this is presented to more clearly demonstrate the existence of these branches as being part of evolutionary pathways if that is indeed the case.*

Response: Yes, addressing this issue resulted in an additional analysis (p. 11), describing two specific regions: the transition and bottleneck regions (see also new Figures 4 and 5, as well as Figures 10, 12 and 14 in SI). The structure of the centrality-prevalence space is now explained in more detail, and is investigated for different clustering thresholds as well (Figures 19 – 22 in SI).

> *227-228: How could a strain "lose its distance genetic relatives" ? If two strains are really related, wouldn't they just become more distant?*

Response: Rephrased, as becoming more distant (line 244).

> *257-263 Do these identified paths fall into one of the two branches or do they tend to cross both branches? It somewhat surprising that so many paths have a net reduction in centrality when eyeballing the distribution of colors and points in figure 3a. Although the density of points can be hard to see.*

> *267-269: In a similar vein, how is one to know that LR-BT chains are actually associated with the explorative branch based on the data shown? In other words, for this data, how can I know what part of the centrality prevalence space plot these chains transect?*

Response: These two questions are now clarified with the additional analysis (p. 11), and a quantitative summary of the transition region is now provided on p. 12. We also estimated some correlations more precisely on pp. 14-15, after removing some incomplete data points (which reduced the number of isolates from 17,107 to 17,005).

Supporting Information

> *I recommend that all this information be moved to materials and methods.*

Response: This is now moved to Materials and Methods, with only supplementary figures left in SI.

395-399: pb should be bp

Response: Fixed.

409-410: Why not allow any locus to vary by more than one tandem repeat here? Logically I would think that if profiles i and j differ by 1 tandem repeat in 1 locus, and 2 tandem repeats in one locus, they should be treated equally to two profiles that differ by 1 tandom repeat in 3 different loci. It also seems strange to me to preclude relatedness of such an example while allowing some relatedness between two profiles that differ by 1 tandom repeat in 5 different loci.

Response: In this work we considered two different distance measures, representing the “extremes” in defining genetic differences: (i) the multiple-loci distance captures dispersed but limited genetic variations across several loci, and (ii) the single-locus distance quantifies “local but variable genetic variations. There are several other ways to define distances, including Manhattan L_1 distance, which we explored in the previous work (and which is used for inferring the undirected network in this study).

423: Additional explanation, or an example, of clustering would be helpful to the reader, since it's unclear from the text as written how exactly the 1,674 unique profiles/nodes gets reduced or transformed into <1,674 clusters.

Response: We slightly restructured Materials and Methods, with Clustering becoming a subsection. It now includes an illustrative example. We also detailed results of clustering with four different thresholds (G_{max}), further exemplifying the concept, and ranging from singleton-clusters (G_{max} = 0) to large overlapping clusters (G_{max} = 10).

425: So each node (i.e. profile) is associated with at least one cluster, even if that cluster only contains a single node?

Response: Yes, this is clarified now (see the previous point).

426: Is Gmax = 5 even when using the method from Larsson et al with 6 loci?

Response: We have now addressed an ambiguity in dealing with Larsson’s correction. Rather than appending the 27bp and 33bp counts for the last locus (that is, appending 2-12, for example), we append their sum, e.g., 14 as the count for the last locus. In other words, there are counts for 5 loci used in our analysis. This is now corrected with G_{max} = 5 used for the main thread of the study.

429-430: Please restate the definition of N.

Response: Restated (line 479).

431: What is the rational for only looking at chains with a length greater than five edges?

Response: We actually included all chains (and the erroneous line is now removed).

Figure 1

Overall I really like this figure and think it is a neat way to represent the data. The figures overall in this manuscript look very professional.
Shouldn't the y-axis be "Unique MLVA profiles", since the MLVA is the analysis itself?

Response: Fixed.

If I'm understanding this right, a single MLVA profile (that is, a single horizontal row) would always be a single "color", if you were to color all case studies uniquely, correct? It's hard to verify this since the rows all slightly overlap, and it's hard to trace a single row with all the white space between individual case-points.

Response: Yes, each row is a single MLVA profile. We tried to make this clearer in the caption.

It would be helpful to the reader to identify exactly what the case studies are of, as it took me until much later in the manuscript to understand that they are the 3 possible evolutionary paths out of the 6,896 potential evolutionary paths, and that this is the number of possible paths because of constraints the authors places on how different "related" strains could be (because in theory all strains share an evolutionary path if you didn't subject them to the limits described in the SI). So pointing out the total possible "case studies" and pointing the reader to the SI section where you define these would be very useful.

Response: These additional details are provided.

("indicating that new MLVA profiles are usually detected regularly throughout the observational period") It's worth adding that new instances of old MLVA profiles also seem to be detected regularly.

Response: Added.

Is there a reason the profiles are ordered oldest at top? It's not super important, but I would think oldest at the bottom makes more sense, since if you wanted to recreate this figure in a year it would extend the top of the figure instead of the bottom of the figure.

Response: This was left as is, with time dimension going down (e.g., following conventions used in cellular automata, etc.).

Can the authors please add labels to the x-axis on the inset?
This data might be better suited to be represented with non-overlapping square shaped points, since each point is a discrete day. It would be a little easier to trace an MLVA profile row this way as well.

Response: We have now used a more conventional way of insetting figures, however did not include labels and different point styles, as alternatives did not make it more clear.

Figure 2

("The network comprises 690 nodes") Please explain in the caption why there aren't there 1,674 nodes here if, as stated in figure 1, there are 1,674 unique MLVA profiles.

Response: There are 690 connected nodes in the directed network, and the rest are disconnected – we added the word "connected".

("each profile may be associated with a cluster of other profiles within a genetic distance G. The size of the nodes represents the average prevalence within this cluster. In this network, the node associated with the most prevalent cluster (profile 3-9-7-17-523) is highlighted and circled in orange.") This part really confused me the first time I read it. What is the purpose of discussing clusters of profiles, or sizing the nodes by average prevalence within a cluster (especially since the reader doesn't know the significance of a cluster or the number of nodes in each cluster here)? Why not just scale the size based on the average profile prevalence? I'm not sure how insightful either really is though, since a profile might span 10 years and be associated with different instances of the strain. Node size here is also relatively imperceptible.

Response: We clarified the process and benefits of clustering in addressing the previous points. The slightly modified caption now refers to Model, as well as Materials and Methods.

How is there only one profile in the most prevalent "cluster" of profiles?

Response: This comment is not immediately relevant to this figure, as the most prevalent overlapping clusters may, in general, have many nodes. Fig. 2 does not show overlapping clusters, but rather network components. The orange circle does not indicate that the reference node is a singleton-cluster, and is used only to highlight the reference node associated with the most prevalent cluster.

(“From this graph, we can capture a potential evolutionary path between two connected nodes as a sequence of directed edges between them.”) Please reference the section where the method for determining an evolutionary path is described here for easy access for the reader.

Response: Added.

(“we illustrate the longest path (17 nodes) inferred from the dataset as a dashed black line with a source node near the top-left of the network”) Please reference the section where the method for determining an evolutionary path is described here for easy access for the reader. It was again a bit confusing here because without the constraint for evolutionary relationship stated, I would think that you should be able to draw a path between all nodes.

Response: Added.

Please include a color-bar for scale

Response: Colour is not strongly relevant for this figure, as different colours are used only to distinguish between paths and components, and we do not mean to highlight a correlation between component size and path lengths in this figure.

Figure 3

As I mentioned in the main text comments, I do not believe the evolutionary trend as stated is clear here in figure 3a, or the step-wise prevalence increases mentioned for 3b left.

3a

Describing this as "The trajectory of evolutionary paths" is confusing because the only trajectories shown are those of the 3 case studies, otherwise all the individual points are just profiles.

Response: This figure is now split, and the caption’s emphasis for new Fig. 3 is now on the structure-function relationship in the genotype networks, mapping the centrality of profiles (x-axis) and their average cluster prevalence (y-axis), rather than on evolutionary trends per se.

So are the grey profiles not included at all in the directed network? Or is it just that they result in singletons/disconnected nodes when they're included?

Response: Grey profiles are disconnected nodes, this is now clarified.

3b

It might be nice if the y-axes here had the same range as 3a, since they're showing the same y-values. On the right plot, something like a violin plot may be more clear since there are many overlapping points, and there is plenty of room within the x-space.

Response: Fig. 3b is now a separate Fig. 6. We opted against using a violin plot, as the connected-component size is a discrete, rather than categorical, variable (i.e., the figure relates to bivariate association, not multivariate distributions).

Figure 4

The manuscript jumps between saying path and chain (e.g. path length is mentioned in the caption but chain length is used in the figures) . If there is no difference in what the authors mean by these words, they should choose only one to use throughout the manuscript to avoid confusion.

Response: Yes, this ambiguity is now removed throughout the text, with the word “path” used consistently (Figure 4 is Fig. 7 now).

Figure 5

Is "Probability" on the y-axes meant as "statistical significance"? If so, I would replace the label so it's clear that lower-values are "better".
I don't quite understand this figure. What are the shaded blue and red triangles showing? Which blue triangle corresponds to the blue arrows and blue dashed lines? What are the solid lines?

Response: Figure 5 is Fig. 8 now (moved to Materials and Methods). We left the y-axis label as “Probability” to reflect the cumulative probability distribution nature of the plots. We also added to the caption an explanation of shaded blue/red triangles.

Thanks again for your consideration.

Yours sincerely,

Prof Mikhail Prokopenko
(on behalf of all co-authors)
The University of Sydney
Sydney, Australia
