# Supplementary material for: Inferring evolutionary pathways and directed genotype networks of foodborne pathogens
Source: PLoS Comput Biol. 2020 Oct 30;16(10):e1008401. doi: 10.1371/journal.pcbi.1008401 (PMC7657559; doi:10.1371/journal.pcbi.1008401)
Supplement: Attachment — Submitted filename: Response 18-Sep-2020.pdf [file pcbi.1008401.s016.pdf]

James Lloyd-Smith 18 September 2020
Associate Editor
PLOS Computational Biology

RE: PCOMPBIOL-D-20-00724

We would like to thank you and the referees for reviewing our paper (2nd round) and providing additional comments. Please find attached a new version of the manuscript, revised according to the 2nd reviewer's suggestions, with the modifications marked in red. Our specific point-by-point responses are below.

Reviewer #2

> *1. The clarified description of the clustering is much better at communicating how the clustering works. However, I feel that this clarity is not properly reflected in Fig. 2. ... To make a minimal concrete suggestion: the sentence "Formally, the cluster Ci associated with node i is given by C i= { j : G ij≤ G max} ," should just be included in the caption.*

Response: We added the suggested explanation to the caption of Fig. 2 (p. 22).

> *2. ...Unfortunately, the 3rd and 4th paragraphs of the results section appear completely unchanged (lines 224-248), and the referenced figure 3 still gives no visible indications or explicit descriptions of where these two "well-defined" branches are, or where the "plateaus" are. ... I believe that communication of the results would be greatly improved if lines 224-248 either forgo reference to the ill-defined branches, or if the branches are made explicit--including their relation to the "transition region"--using either visual cues/text on the figure, and/or additional explicit main-text description.*

Response: The 3rd paragraph on p. 10 is extended with a detailed description of the branches. The caption of Fig. 3 (p. 23) is extended accordingly.

Thanks again for your consideration.

Yours sincerely,

Prof Mikhail Prokopenko
(on behalf of all co-authors)
The University of Sydney
Sydney, Australia
